# Crosstalk between insulin-like growth factor-1 and angiotensin-II in dopaminergic neurons and glial cells: role in neuroinflammation and aging

**DOI:** 10.18632/oncotarget.9174

**Published:** 2016-05-04

**Authors:** Ana I. Rodriguez-Perez, Ana Borrajo, Carmen Diaz-Ruiz, Pablo Garrido-Gil, Jose L. Labandeira-Garcia

**Affiliations:** ^1^ Laboratory of Neuroanatomy and Experimental Neurology, Department of Morphological Sciences, CIMUS, University of Santiago de Compostela, Santiago de Compostela, Spain; ^2^ Networking Research Center on Neurodegenerative Diseases (CIBERNED), Madrid, Spain

**Keywords:** IGF-1, longevity, microglia, neurodegeneration, Parkinson, Gerotarget

## Abstract

The local renin-angiotensin system (RAS) and insulin-like growth factor 1 (IGF-1) have been involved in longevity, neurodegeneration and aging-related dopaminergic degeneration. However, it is not known whether IGF-1 and angiotensin-II (AII) activate each other. In the present study, AII, via type 1 (AT1) receptors, exacerbated neuroinflammation and dopaminergic cell death. AII, via AT1 receptors, also increased the levels of IGF-1 and IGF-1 receptors in microglial cells. IGF-1 inhibited RAS activity in dopaminergic neurons and glial cells, and also inhibited the AII-induced increase in markers of the M1 microglial phenotype. Consistent with this, IGF-1 decreased dopaminergic neuron death induced by the neurotoxin MPP^+^ both in the presence and in the absence of glia. Intraventricular administration of AII to young rats induced a significant increase in IGF-1 expression in the nigral region. However, aged rats showed decreased levels of IGF-1 relative to young controls, even though RAS activity is known to be enhanced in aged animals. The study findings show that IGF-1 and the local RAS interact to inhibit or activate neuroinflammation (i.e. transition from the M1 to the M2 phenotype), oxidative stress and dopaminergic degeneration. The findings also show that this mechanism is impaired in aged animals.

## INTRODUCTION

Insulin-like growth factor 1 (IGF-1) has been implicated in life-span and in many other functions, including several brain functions [1, 2]. For many years, IGF-1 was considered a cytoprotective factor. However, it has also been suggested that IGF-1 may be detrimental to health, and that reduced IGF-1 levels lead to prolonged life [3, 4]. The effects of IGF-I in the brain and particularly in the aged brain are unclear [5–7]. This is further complicated by the dual origin of the brain IGF-1. IGF-1 crosses the blood brain barrier, possibly *via* the choroid plexus [8]. Local IGF-1 is also produced by neurons and glial cells [9, 10], and the role of this local IGF-1 remains to be clarified. A reduction in IGF-1 activity with age has been related to the pathogenesis of age-related neurodegenerative diseases [11, 12]. The role of IGF-1 in the pathogenesis of Parkinson's disease (PD) is puzzling. The substantia nigra is one of the brain regions with highest density of IGF-1 receptors (IGF-1R) [9], and IGF-1 has been found to increase survival of dopaminergic neurons both *in vitro* and in animal models of PD [13, 14]; however, increased levels of IGF-1 have been observed in the serum and cerebrospinal fluid of PD patients, which has been suggested a as possible marker for risk and early diagnosis of PD [15, 16].

Angiotensin II (AII) is the most important effector peptide of the renin-angiotensin system (RAS). Its effects are mediated by two main cell receptors: AII type 1 and 2 (AT1 and AT2) receptors. AT2 receptors exert actions that are directly opposed to those mediated by AT1 receptors [17, 18]. Hyperactivation of local or tissue RAS, *via* AT1 receptors and NADPH oxidase activation, mediates oxidative stress (OS) and several key events in inflammatory processes and has been associated with decreased longevity and age-related degenerative changes in a number of tissues [19–21]. NADPH-oxidase is the second source of OS after mitochondria [22]; in addition, NADPH-derived ROS (reactive oxygen species) interact with mitochondria to increase levels of OS in dopaminergic neurons and other cells [23–25]. The brain has a local or tissue RAS that is independent of the circulating RAS and in which astrocytes constitute the major source of the precursor protein angiotensinogen [26, 27]. In recent studies, we have demonstrated the presence of local RAS in the substantia nigra and striatum of rodents and primates [28–30], including humans [31]. It has also been demonstrated that overactivation of local RAS, *via* AT1 receptors, exacerbates neuroinflammation, OS and dopaminergic cell death [23, 28, 32, 33]. Overactivation of RAS has been observed in the nigrostriatal system of aged rats, along with increased dopaminergic cell vulnerability to neurotoxins [34, 35] and possibly PD [26, 27, 36].

The local RAS and IGF-1 both are involved in neuroinflammation, OS and aging-related dopaminergic vulnerability to damage. However, it is not known whether IGF-1 and AII inhibit or activate each other during these processes; clarification of this interaction may be useful for the development of neuroprotective and anti-aging treatments [37]. In the present study, we used *in vitro* (primary mesencephalic cultures and dopaminergic and microglial cell lines) and *in vivo* (young and old rats) models to investigate the possible reciprocal regulation between IGF-1 and AII in the dopaminergic system.

## RESULTS

### Location of IGF-1 and IGF-1R in dopaminergic neurons and glial cells

**Figure 1:**
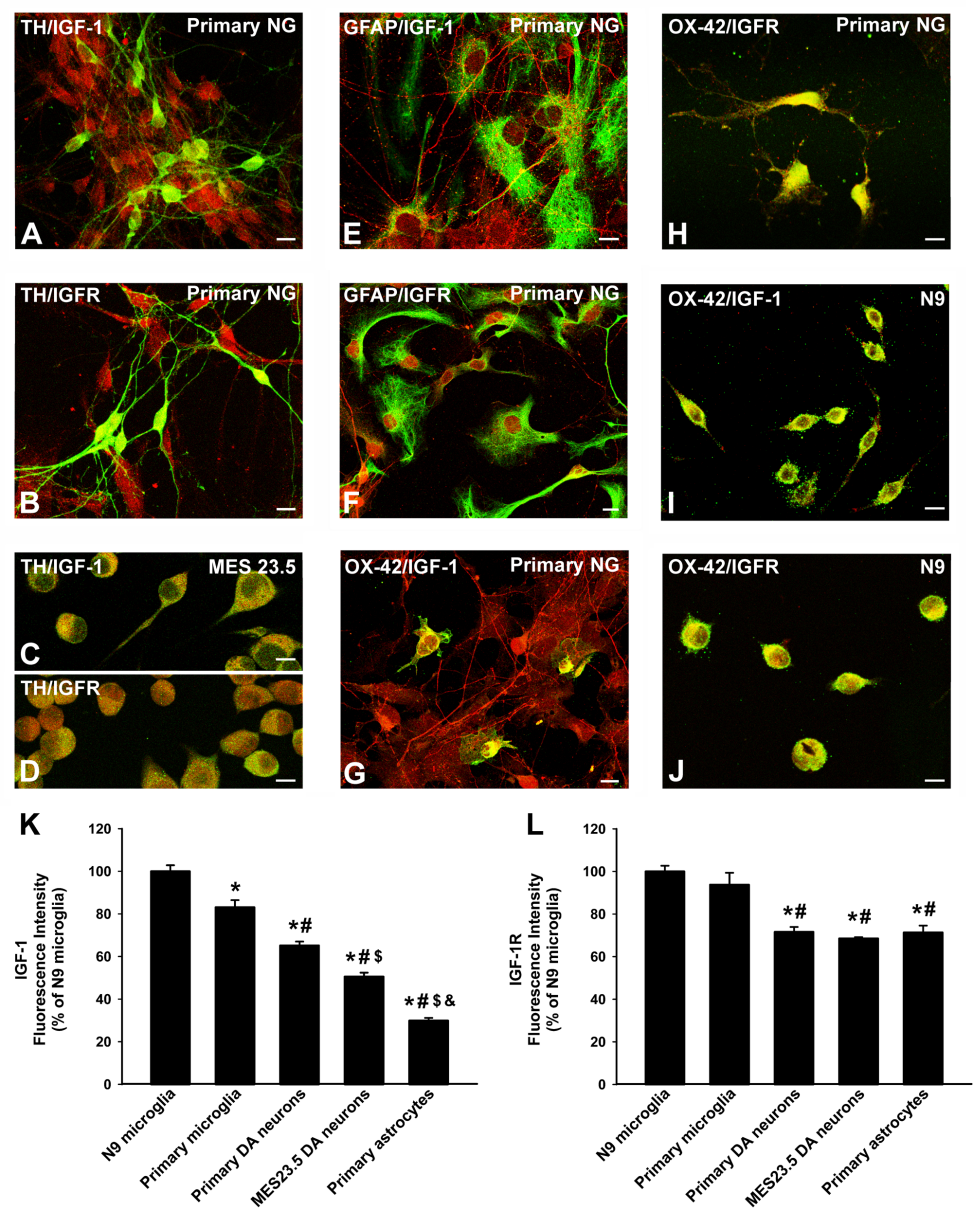
Localization of IGF-1 and IGF1receptors in dopaminergic neurons and glial cells Double immunofluorescence and laser confocal microscopy showing co-localization (yellow) of the dopaminergic marker TH (green; **A.**-**D.**, the astroglial marker GFAP (green), **E.**, **F.** or the microglial marker OX-42 (green), **G.**-**J.**, and IGF-1 (red), **A.**, **C.**, **E.**, **G.**, **I.** or IGF-1R (red), **B.**, **D.**, **F.**, **H.**, **J.** Dopaminergic neurons in primary cultures and MES 23.5 dopaminergic neurons showed immunopositivity for IGF-1 **A.**, **C.** and IGF-1R **B.**, **D.**. Immunolabelling for IGF-1 and IGF-1R was also detected in primary astrocytes **E.**, **F.** and more intense immunolabeling for both IGF-1 and IGF-1R was observed in primary microglia **G.**, **H.** and the N9 microglial cell line **I.**, **J.**. The relative intracellular levels of IGF-1 and IGF-1R was estimated by computer-assisted fluorescence intensity measurements **K.**, **L.**. Data represent means ± SEM. *p < 0.05 compared with control group (N9 microglial cells); ^#^*p* < 0.05 relative to primary microglia; ^$^*p* < 0.05 relative to primary dopaminergic (DA) neurons; ^&^*p* < 0.05 relative to MES 23.5 DA neurons. One-way ANOVA and Holm Sidak post-hoc test. Scale bars: 10 μm. GFAP, glial fibrillary acid protein; IGF-1-R, IGF-1 receptor; TH, tyrosine hydroxylase; primary NG, primary neuron-glia mesencephalic cultures.

Primary dopaminergic neurons and MES 23.5 dopaminergic neurons showed immunopositivity for IGF-1 (Figure 1A, 1C) and more marked immunolabeling for IGF-1R (Figure 1B, 1D). Immunolabeling for IGF-1 and IGF-1R was also detected in astrocytes in both primary neuron-glia cultures and in astrocyte-enriched cultures (Figure 1E, 1F). IGF-1 and IGF-1R immunolabelling was also observed in primary microglia (Figure 1G, 1H) and the N9 microglial cell line (Figure 1I, 1J), which showed the most intense immunolabeling (Figure 1K, 1L).

### Neuroprotective effect of IGF-1 against MPP^+^ neurotoxicity

**Figure 2:**
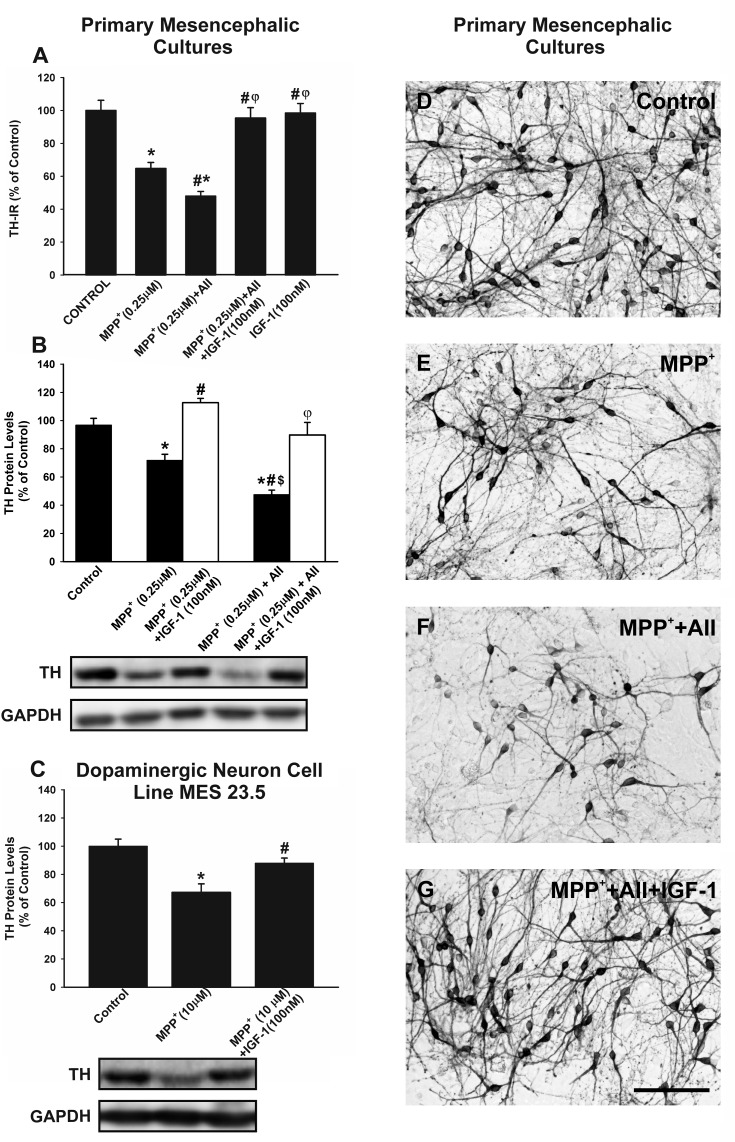
Effects of treatment with AII and IGF-1 on MPP+-induced neurotoxicity in dopaminergic (TH-ir) neurons In primary cultures, treatment with 0.25 μM MPP^+^ induced a significant loss of TH-ir neurons **A.**, **D.**-**E.** and expression of TH protein **B.**, which was significantly increased by AII (100 nM) and inhibited by IGF-1 (100 nM) **A.**, **B.**, **D.**-**G.**. A direct protective effect of IGF-1 on TH-expression (i.e. not mediated by effects on glial cells) was shown by treating the dopaminergic neuron cell line MES 23.5 with MPP^+^ in the presence or the absence of IGF-1 **C.** The number of TH-ir neurons is expressed as a percentage of the number of TH-ir cells obtained in the respective control cultures (100%). Protein expression was measured relative to the GAPDH band value. The results were normalized to the values for controls (100%). Data represent means ± SEM. *p < 0.05 compared with control group (untreated cells); ^#^*p* < 0.05 relative to the group treated with MPP^+^; ^φ^p < 0.05 relative to MPP^+^ + AII; ^$^*p* < 0.05 relative to MPP^+^ + IGF-1. One-way ANOVA and Holm Sidak post-hoc test. Scale bars: 100 μm. AII, angiotensin II; TH, tyrosine hydroxylase.

In primary mesencephalic cultures, treatment with IGF-1 (100 nM) inhibited the loss of tyrosine hydroxylase-immunoreactive (TH-ir) neurons induced by treatment with the dopaminergic neurotoxin MPP^+^ (0.25 μM). As observed in previous studies, the effect of MPP^+^ was significantly enhanced by administration of AII (100nM), which was also inhibited by treating cultures with IGF-1 (Figure 2A, 2D-2G). We confirmed the neuroprotective effects of IGF-1 against MPP^+^ and MPP^+^ plus AII by using WB to quantify TH protein levels (Figure 2B). The possible direct protective effect of IGF-1 on neurons (i.e. not mediated by effects on glial cells) was investigated by treating the dopaminergic neuron cell line with MPP^+^ in the presence or absence of IGF-1. As observed in previous studies, the doses of MPP^+^ required to induce a similar loss of neurons was much higher (10 μM) in the absence of glia than in primary neuron-glia cultures. For details see [28, 29, 38]. The loss of TH expression induced by MPP^+^ was also inhibited by co-treatment with IGF-1 in the absence of glia (Figure 2C)

### Effect of IGF-1 on RAS activity

**Figure 3:**
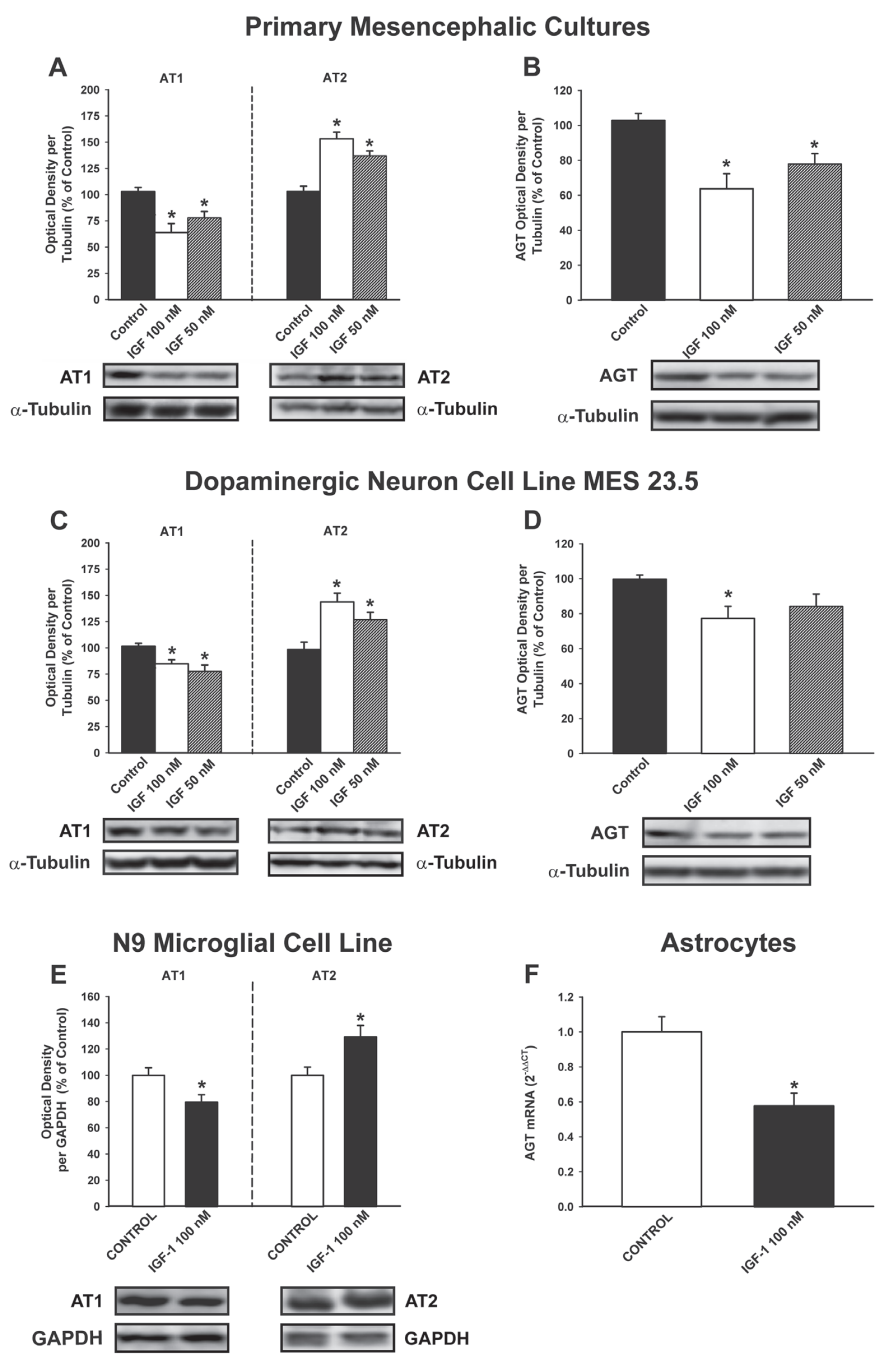
Effect of treatment with IGF-1 (50 or 100 nM) on the renin-angiotensin system (RAS) Western blot analysis **A.**-**E.** and real-time quantitative RT-PCR analysis **F.** of changes induced in the expression of angiotensin receptors (AT1 and AT2) and angiotensinogen (AGT) in primary (neuron-glia) mesencephalic cultures **A.**, **B.**, the dopaminergic cell line MES 23.5 **C.**, **D.**, the N9 microglial cell line **E.** and primary astroglial cultures **F.** Protein expression was measured relative to the α-tubulin or GAPDH band value. The results were normalized to the values for controls (100%). For RT-PCR the comparative cycle threshold values method (2^−ΔΔCt^) was used. Expression of the AGT gene was measured relative to that of the housekeeping transcripts (β-Actin). Data are means ± SEM. *p < 0.05 relative to controls. One-way ANOVA followed by Holm Sidak post-hoc test **A.**-**D.** and Student's t test **E.**, **F.**

We performed *in vitro* studies to investigate the possible effects of IGF-1 on RAS activity, and to identify the types of cells involved in these effects. WB studies in primary mesencephalic cultures revealed that treatment with IGF-1 induced a significant decrease in AT1 receptor expression, a significant increase in the expression of AT2 receptors (i.e. a marked decrease in the AT1 to AT2 ratio), and decreased expression of angiotensinogen (Figure 3A, 3B). A similar decrease in RAS activity (i.e. decreased AT1 expression, increased AT2 expression and decreased angiotensinogen levels) was observed after treatment of the dopaminergic neuron cell line MES 23.5 (i.e. in the absence of glia) (Figure 3C, 3D). Treatment of the N9 microglial cell line with IGF-1 also induced downregulation of AT1 receptor expression and upregulation of AT2 receptor expression (Figure 3E; see also Figure 8D). Astrocytes constitute the major source of angiotensinogen in the CNS. Using primary astrocyte-enriched cultures, we observed that treatment with IGF-1 induced a significant decrease in angiotensinogen levels. This was confirmed by RT-PCR, which showed that the regulatory changes occurred at transcriptional level (Figure 3F), and was consistent with changes observed in primary neuron-glia cultures (Figure 3B).

### Effect of RAS activity on IGF-1 expression

**Figure 4:**
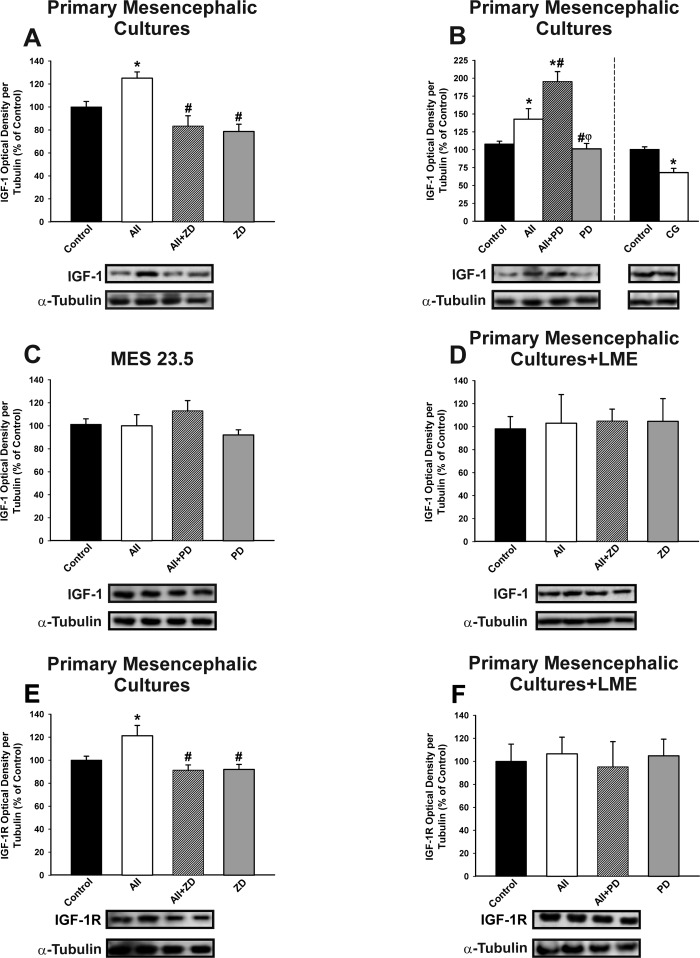
Effect of treatment with AII (100 nM) on IGF-1 (A-D) and IGF-1 receptor (IGF-1R; E-F) Western blot analysis of changes induced by treatment with AII in expression of IGF-1 and IGF-1R in primary mesencephalic cultures **A.**, **B.**, **E.**, the dopaminergic neuron cell line MES 23.5 **C.** and primary mesencephalic cultures lacking microglial cells (i.e. treated with LME) **D.**, **F.** The AII-induced increase in IGF-1 and IGF-1R expression was inhibited by the AT1 receptor antagonist ZD-7155 and the AT2 agonist CG-42112A, and enhanced by the AT2 receptor antagonist PD-123319. However, treatment with AII did not induce significant changes in IGF-1 and IGF-1R in the absence of microglia **C.**, **D.**, **F.** Protein expression was measured relative to the α-tubulin band value. The results were normalized to the values for controls (100%). Data are means ± SEM. *p < 0.05 relative to controls; ^#^*p* < 0.05 relative to AII-treated group; ^φ^*p* < 0.05 relative to AII+PD group. One-way ANOVA followed by Holm Sidak post-hoc test. AII, angiotensin II; CG, AT2 agonist CG-42112A; LME, L -leucine methyl ester; PD, AT2 antagonist PD-123319; ZD, AT1 antagonist ZD-7155.

Treatment of primary mesencephalic (neuron-glia) cultures with AII induced a discrete but significant increase in levels of IGF-1, which was blocked by treatment with the AT1 receptor antagonist ZD-7155 (Figure 4A). Interestingly, IGF-1 levels in primary cultures increased further after treatment of cultures with the AT2 receptor antagonist PD-123319 (i.e. AII+PD). The results show that AII induces IGF-1 in cultures *via* the AT1 receptor, and that this effect is inhibited by AT2 receptors. The inhibitory effect of AT2 receptors on AII-induced expression of IGF-1 was confirmed by treating cultures with the AT2 agonist CG-42112A (Figure 4B).

Treatment of the dopaminergic neuron cell line with AII and/or AII receptor antagonists did not induce any significant change in IGF-1 levels, suggesting that the effects of AII on IGF-1 observed in primary cultures mainly involved glial cells (Figure 4C). Additional series of primary mesencephalic cultures were treated with L-leucine methyl ester (LME) to remove the microglial cells, and they were then treated with AII. Again, no significant changes in IGF-1 levels were induced by AII, indicating that microglial cells are responsible for the IGF-1 upregulation induced by AII in neuron-glia cultures (Figure 4D). Treatment of neuron-glia primary cultures with AII also induced a significant increase in levels of IGF-1R, which was not observed in the absence of microglia (i.e. cultures treated with LME), suggesting that the increase in IGF-1R mainly involves microglial cells (Figure 4E, 4F).

**Figure 5:**
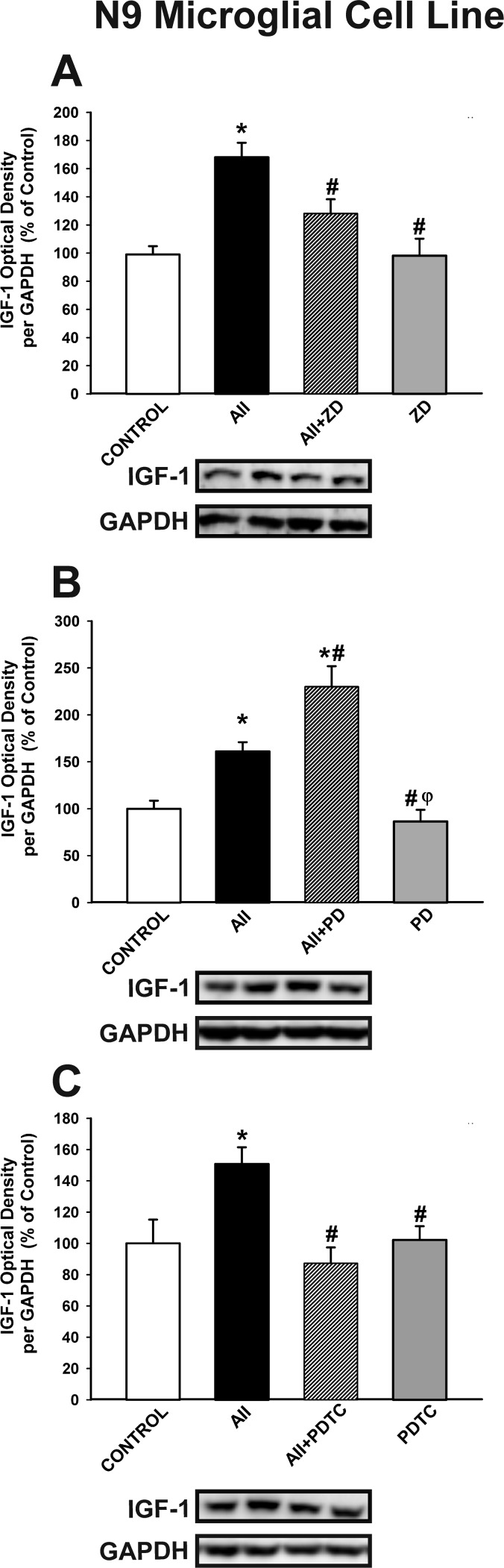
Western blot analysis of the effects of AII on IGF-1 expression in N9 microglial cells The increase induced by AII (100 nM) in IGF-1 expression was inhibited by the AT1 receptor antagonist ZD-7155 **A.** and the NF-κB inhibitor PDTC **C.**, and enhanced by the AT2 receptor antagonist PD-123319 **B.** Protein expression was measured relative to the GAPDH band value. The results were normalized to the values for controls (100%). Data are means SEM. *p < 0.05 relative to controls; ^#^*p* < 0.05 relative to AII-treated group; ^φ^*p* < 0.05 relative to AII+PD group. One-way ANOVA followed by Holm Sidak post-hoc test AII, angiotensin II; PDTC, NF-κB inhibitor ammonium pyrrolidinedithiocarbamate; PD, AT2 antagonist PD-123319; ZD, AT1 antagonist ZD-7155.

The effect of AII on microglial IGF-1 levels was confirmed in the N9 microglial cell line. Treatment of N9 microglial cells with AII induced a significant increase in levels of IGF-1, which was blocked by treatment with the AT1 receptor antagonist ZD-7155 (Figure 5A). As observed in neuro-glia cultures, IGF-1 levels were further increased by treating cultures with the AT2 receptor antagonist PD-123319 (Figure 5B). Interestingly, the AII-induced increase in microglial IGF-1 levels was blocked by treatment with the NF-κB inhibitor PDTC (ammonium pyrrolidinedithiocarbamate) (Figure 5C), which indicates that translocation of NF-κB mediates the effect of the AII/AT1 axis on levels of microglial IGF-1

**Figure 6:**
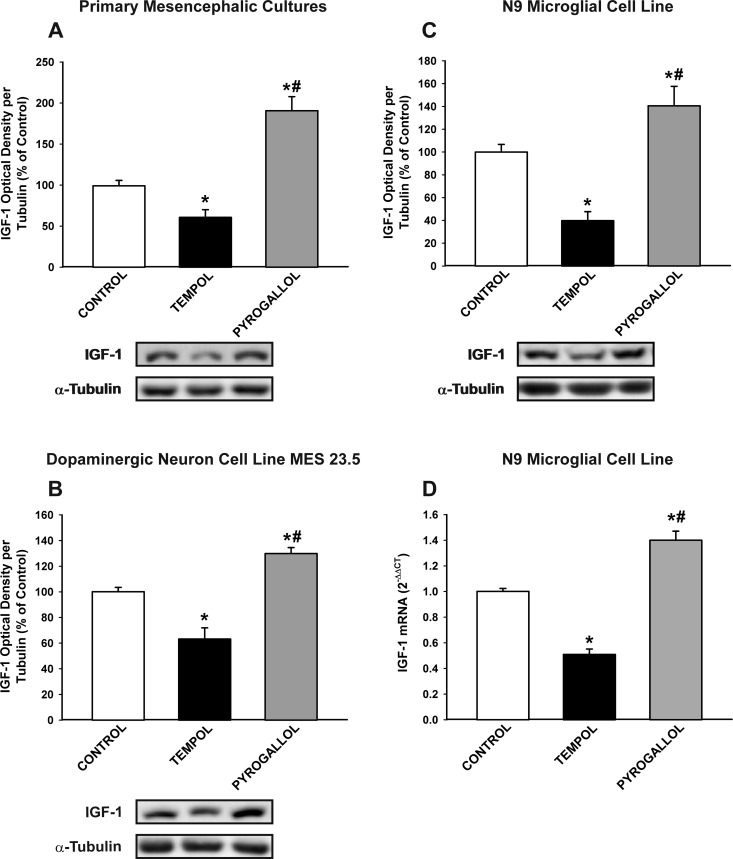
Effect of the pro-oxidant compound pyrogallol and the anti-oxidant tempol in the expression of IGF-1 Western blot analysis **A.**-**C.** and real-time quantitative RT-PCR analysis **D.** of changes induced by pyrogallol and tempol in primary mesencephalic cultures **A.**, the dopaminergic cell line MES 23.5 **B.** and the N9 microglial cell line **C.**, **D.** The anti-oxidant tempol induced a significant decrease in IGF-1 expression and the pro-oxidant pyrogallol induced a significant increase in IGF-1 expression relative to the controls. Protein expression was measured relative to the α-tubulin band value. The results were normalized to the values for controls (100%). For RT-PCR, the comparative cycle threshold values method (2^−ΔΔCt^) was used. The IGF-1 gene expression was measured relative to that of the housekeeping transcripts (β-Actin). Data are means ± SEM. *p < 0.05 relative to controls; ^#^*p* < 0.05 relative to the tempol-treated group. One-way ANOVA followed by Holm Sidak post-hoc test.

As the AII/AT1/NADPH-oxidase axis is a major source of OS in cells, particularly in microglial cells, we investigated the effect of the pro-oxidant compound pyrogallol and the antioxidant compound tempol on IGF-1 levels in neurons and glial cells. Treatment with pyrogallol induced a significant increase in IGF-1 levels in primary (neuron-glia) cultures, dopaminergic neuron cell line MES 23.5 cultures, and N9 microglial cell line cultures (Figure 6A-6D). This indicates that the dopaminergic neurons also upregulate IGF-1 to counteract OS, and that levels of OS induced by AII in neurons in the absence of microglia (at least in the present experimental conditions; Figure 4C-4D) do not induce a detectable increase in neuronal IGF-1. In concordance with this, treatment of cultures (primary cultures, MES 23.5 dopaminergic neuronal cell line and N9 microglial cell line) with the antioxidant tempol significantly reduced the expression of IGF-1.

### Effect of AII and IGF-1 on major markers of the microglial M1 and M2 phenotypes

**Figure 7:**
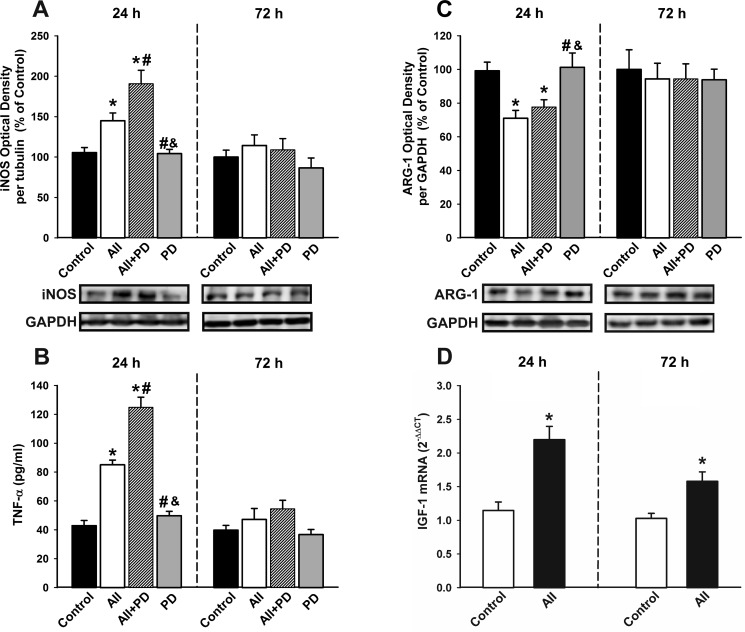
Changes induced by treatment with AII in the expression of markers of the microglial phenotype Western blot **A.**, **C.**, ELISA **B.** and real-time quantitative RT-PCR **D.** of effects AII (100 nM) in the expression of the markers of the M1 cytotoxic phenotype (iNOS and TNF-α ; A, B), the marker of the M2 repair/regenerative phenotype ARG-1 **C.**, and IGF-1 **D.** in N9 microglial cells 24 and 72h after treatment. Protein expression was measured relative to the GAPDH band value. The results were normalized to the values for controls (100%). TNF-α levels were expressed in pg/ml protein. For RT-PCR, the comparative cycle threshold values method (2^−ΔΔCt^) was used. The IGF-1 gene expression was measured relative to that of the housekeeping transcripts (β-Actin). Data are means ± SEM. *p < 0.05 relative to controls; ^#^*p* < 0.05 relative to AII group; ^&^*p* < 0.05 relative to AII+PD group. One-way ANOVA followed by Holm Sidak post-hoc test **A.**-**C.** and Student's *t* test **D.** AII, angiotensin II; ARG-1, arginase-1; iNOS, *inducible nitric oxide synthase;* PD, AT2 antagonist PD-123319; *TNF-α,* tumor necrosis factor alpha.

Treatment of N9 microglial cells with angiotensin induced a significant and early (i.e. 24 h after treatment) increase in the expression of markers of the M1 cytotoxic phenotype including iNOS and TNF-α, which had decreased 72h after treatment. The AII-induced increase in levels of M1 markers such as iNOS and TNF-α was further increased by simultaneous treatment with the AT2 receptor antagonist PD-123319, suggesting that AT2 receptors inhibit this process. Treatment with AII also decreased the expression of markers of the M2 repair/regenerative phenotype such as ARG-1 (Figure 7A-7C). As indicated above (see Figure 5), AII induced an early increase in levels of IGF-1 protein expression in microglial cells, which was still observed 72 h after treatment, and was also shown at the mRNA level by RT-PCR (Figure 7D).

**Figure 8:**
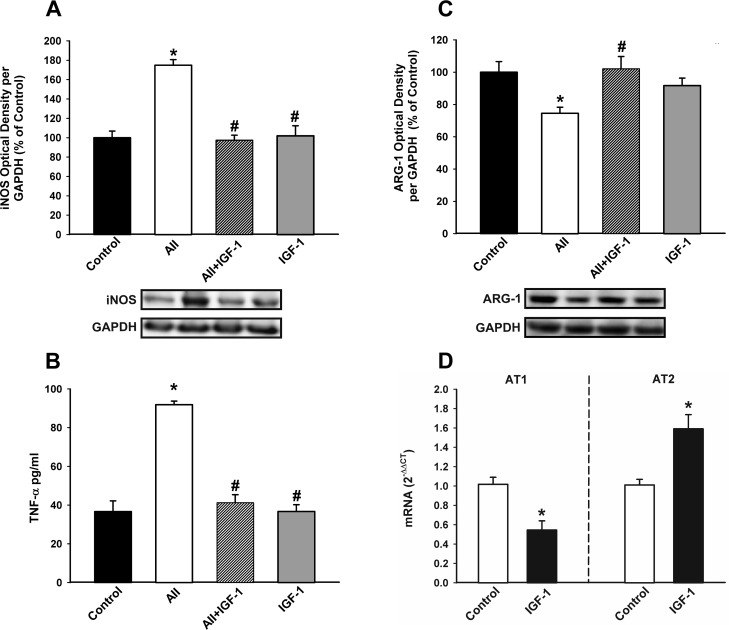
Effect of IGF-1 on AII-induced changes in the expression of markers of the microglial phenotype Western blot **A.**, **C.**, ELISA **B.** and Real-time quantitative RT-PCR **D.** of effects of IGF-1 (100 nM) on the AII-induced changes in expression of markers of the M1 cytotoxic phenotype (iNOS and TNF-α); **A.**, **B.**, a marker of the M2 repair/regenerative phenotype (ARG-1) **C.**, and the expression of angiotensin receptors (AT1 and AT2); **D.** in N9 microglial cells 24 h after treatment. Protein expression was measured relative to the GAPDH band value. The results were normalized to the values for controls (100%). TNF-α levels were expressed in pg/ml protein. For RT-PCR the comparative cycle threshold values method (2^−ΔΔCt^) was used. IGF-1 gene expression was measured relative to that of the housekeeping transcripts (β-Actin). Data are means ± SEM. *p < 0.05 relative to controls; ^#^*p* < 0.05 relative to AII-treated group. One-way ANOVA followed by Holm Sidak post-hoc test **A.**-**C.** and Student's *t* test **D.** AII, angiotensin II; ARG-1, arginase-1; iNOS, *inducible nitric oxide synthase; TNF-α,* tumor necrosis factor alpha.

Treatment of microglial cells with IGF-1 revealed that IGF-1 blocks the AII-induced increase in markers of the M1 cytotoxic phenotype such as TNF- α and iNOS, and the AII-induced decrease in markers of the M2 repair/regenerative phenotype such as ARG-1 (Figure 8A-8C). Consistent with this, IGF-1 decreased the activity of the AII/AT1 axis in microglial cells (i.e. decrease in AT1 receptor expression and increase in AT2 receptor expression; see Figure 3E), which was also shown at mRNA levels by RT-PCR (Figure 8D)

### Effect of RAS overactivation on IGF-1 levels in young and aged rats

**Figure 9:**
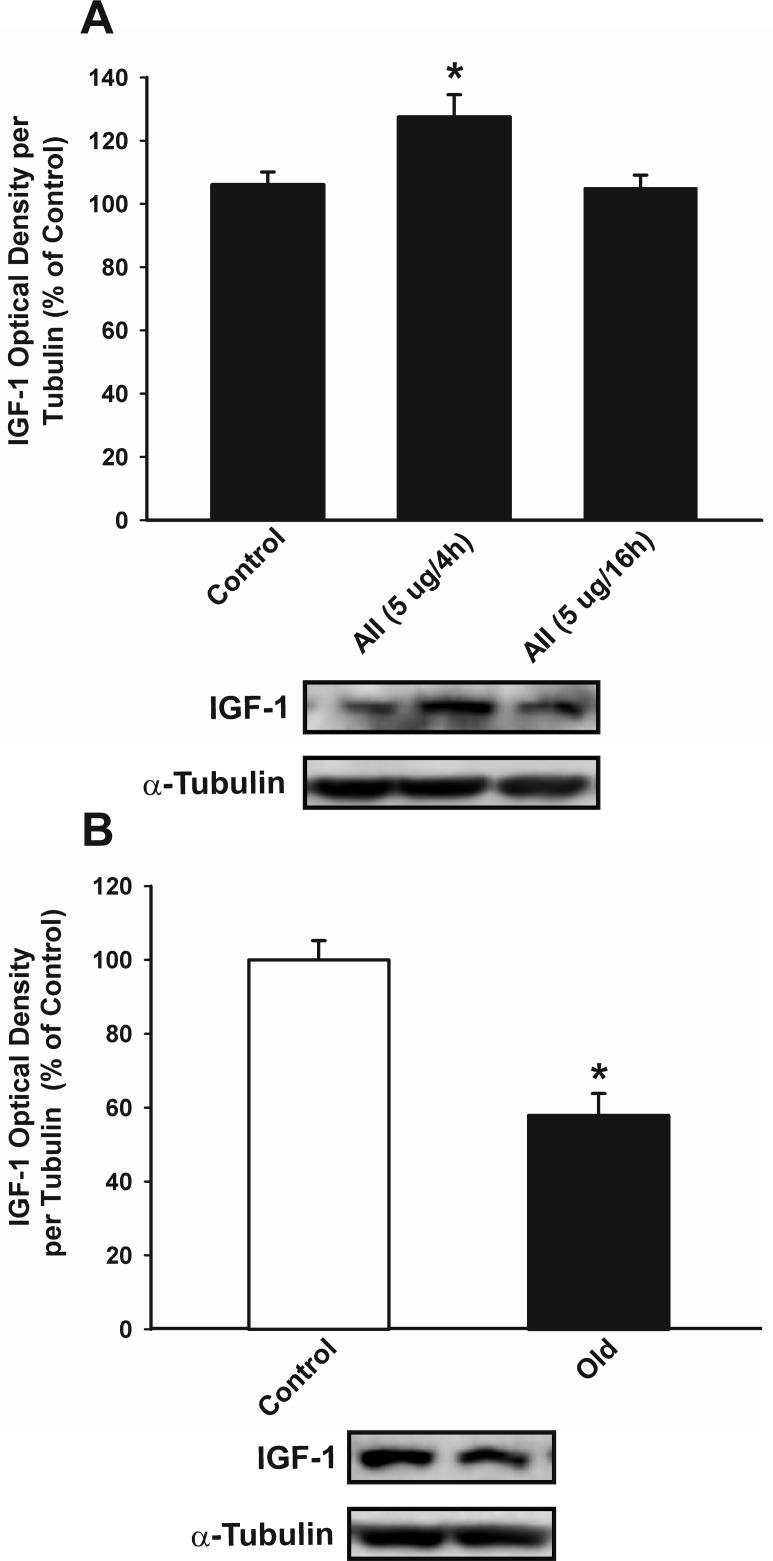
Effect of intraventricular injection of AII in IGF-1 expression in the nigral region of rats Western blot analysis of changes in IGF-1 expression in the nigral region in young adult rats 4 and 16 h after intraventricular injection of AII or vehicle **A.**, and in aged rats relative to controls (i.e. young) rats **B.** Protein expression was measured relative to the α-tubulin band value. The results were normalized to the values for controls (100%). Data are means ± SEM. *p < 0.05 relative to controls. One-way ANOVA followed by Holm Sidak post-hoc test **A.** and Student's *t* test **B.** AII, angiotensin II.

Intracerebroventricular treatment of young adult rats with AII induced a moderate but significant increase in IGF-1 levels in the substantia nigra region 4 h after injection, which was no longer detected 16 h after the injection (Figure 9A). Activation of the AII/AT1/NADPH-oxidase axis is known to be enhanced in the substantia nigra of aged rats; for details, see [34]. However, IGF-1 levels were significantly lower in aged rats than in the young controls (Figure 9B).

## DISCUSSION

In the present study, we confirmed the expression of IGF-1 and IGF-1R in dopaminergic neurons, astrocytes and microglial cells. We also observed that IGF-1 protects dopaminergic neurons from the neurotoxin MPP^+^ both in the presence (i.e. in neuron-glial cultures) and in the absence (MES 23.5 dopaminergic neuron cell line) of glia. A neuroprotective effect on dopaminergic neurons has previously been observed [13, 14]. However, the mechanisms involved in the protective effect have not yet been clarified. Inhibitory effects on the neuroinflammatory response [39], improvement in mitochondrial function [40], inhibition of OS and Sirtuin-1 activation [41, 42] have been suggested as possible mechanisms. Interestingly, the brain RAS has been shown to be involved in neuroinflammation [38, 43], mitochondrial function [23, 24] and Sirtuin-1 activity [44] in PD models. The results of the present study show a reciprocal regulation between IGF-1 and RAS: IGF-1 inhibited RAS activity in neurons and glial cells (i.e. it decreased AT1, increased AT2 and decreased angiotensinogen expression); conversely, AII -*via* AT1 receptors- increased the levels of IGF-1 in microglial cells, while activation of AT2 receptors decreased IGF-1 levels.

### Effects of IGF-1 on RAS activity

It is known that AII, *via* AT1 receptors, activates the NADPH-oxidase complex and increases levels of OS in dopaminergic neurons and microglial cells; for a review, see [26, 36]. In microglia and other inflammatory cells, activation of NADPH-oxidase induces high levels of superoxide, Rho kinase activation and release of inflammatory cytokines as a basic means of the inflammatory response [38, 43]. In neurons and non-inflammatory cells, activation NADPH-oxidase induces low levels of ROS for signaling functions [22]. In the present study, we observed that an increase in IGF-1 levels induced a decrease in RAS activity in mesencephalic neuron-glia cultures (i.e. decreased AT1, increased AT2, and decreased angiotensinogen expression). The decrease in RAS activity took place in dopaminergic neurons (MES 23.5 neuron cell line) and microglia (N9 cell line) and there was also a decrease in the expression of angiotensinogen in astrocytes. Therefore, an increase in IGF-1 levels may directly cause a decrease in levels of ROS in dopaminergic neurons and, more importantly, may indirectly lead to a decrease in neuronal OS by inhibition of the microglial inflammatory response.

### Effects of RAS on IGF-1 expression

The present findings showed that in primary (neuron-glia) mesencephalic cultures AII, *via* AT1 receptors, increases the levels of IGF-1 and IGF-1R in microglial cells, while activation of AT2 receptors decreases IGF-1 and IGF-1R levels. We did not detect any significant increase in IGF-1 levels after treating MES 23.5 dopaminergic neurons with AII. As we have shown (both in the present and previous studies) that MES 23.5 cells have AT1 and AT2 receptors, the present findings suggest that AII cannot induce a significant increase in IGF-1 in neurons, at least under the experimental conditions used, and that microglial cells constitute the origin of the AII-induced IGF-1. This was corroborated by the fact that the AII-induced increase in IGF-1 was not detected in primary cultures lacking microglial cells and is consistent with previous studies suggesting that microglial cells are a major source of brain IGF-1 [10, 45].

IGF-1 was also induced by treating cultures with the pro-oxidant compound pyrogallol, and IGF-1 levels were significantly decreased by treatment with the antioxidant tempol. This was observed in primary (neuron-glia) cultures and the N9 microglial cell line. Interestingly, pyrogallol-induced OS caused an increase in IGF-1 levels in the MES 23.5 neuronal cell line, but this was not observed after treatment with AII. This suggests that AII activates the AT1/NADPH-oxidase axis and induces high levels of ROS in microglia but low levels of ROS in non-inflammatory cells such as neurons; in neurons, AII-induced ROS are not sufficient to induce detectable levels of IGF-1. Nonetheless, higher levels of OS such as those induced by pyrogallol trigger a protective response in neurons that involves IGF-1 induction.

### Reciprocal regulation of AII and IGF-1 in microglial cells. Effect of IGF-1 on the microglial phenotype

The above-mentioned experiments revealed microglia as the major source of the AII-induced IGF-1 and that IGF-1 inhibited RAS activity (decreased AT1 expression and increased AT2 expression) in microglial cells. We, therefore, investigated the effect of AII and IGF-1 on major markers of the microglial M1 and M2 phenotypes [46, 47]. Consistent with previous findings, treatment with AII induced the expression of major markers of the M1 cytotoxic phenotype such as iNOS and TNF-α and decreased markers of the M2-repair/regenerative phenotype such as arginase-1 (ARG-1). The effect of AII was blocked by AT1 antagonists and enhanced by the AT2 antagonists (i.e. the AII-induced enhancement of the M1 phenotype was mediated by AT1 receptors and inhibited by AT2 receptors). Interestingly, the AII-induced increase in markers of the M1 phenotype was blocked by treatment with IGF-1, suggesting that induction of microglial IGF-1 by AII and other OS and pro-inflammatory inducers may play a major role in repressing the M1-neurotoxic phenotype and transition to an M2-repair/regenerative phenotype.

### Effects of RAS activation on IGF-1 expression in young and aged rats

In concordance with the results observed *in vitro*, intraventricular administration of AII to young rats induced a significant increase in IGF-1 expression in the nigral region four hours after the AII injection. However, we observed a decrease in levels of IGF-1 in aged rats relative to the young controls even though RAS activity and markers of OS and neuroinflammation are known to be enhanced in aged rats [32, 34, 35]; a counterregulatory increase in IGF-1 levels may be expected, as observed in young rats. A decrease in IGF-1 levels with aging has also been observed in serum, brain and other tissues [5, 7]. The loss of the inhibitory mechanism of IGF-1 on AT1/NADPH-oxidase described in the present study suggests that the pro-oxidative and pro-inflammatory state that characterizes the aged brain and particularly the aged substantia nigra [32, 34] may be related to low levels of IGF-1 and the loss of capacity of microglia to undergo M2 activation. Interestingly, increased levels of IGF-1 were observed in the serum and cerebrospinal fluid of PD patients, which has been proposed as a possible marker for risk and early diagnosis of PD [15, 16]. This effect may be related to the initial increase in IGF-1 as a neuroprotective mechanism in a relatively young brain against the neuroinflammation and OS that characterize early states of the disease [48]. The effect may be absent in aged brains, which would result in increased vulnerability to development and progression of PD. It is puzzling that a number of studies suggest that IGF-1 is detrimental to health and that low serum and tissue levels of IGF-1 are related to prolonged life [3, 4]. However, it is known that other key factors are also involved in aging and age-related pathologies, such as mTOR (mammalian or mechanistic target of rapamycin) [49, 50] and several mechanisms triggered by caloric restriction (CR) [51, 52], which probably have complex interactions with IGF-1 [49, 51] and RAS [37] in different tissues or specific situations.

In conclusion, the present findings show that IGF-1 and the local RAS interact to inhibit or activate neuroinflammation, OS and dopaminergic degeneration. The results also suggest that this important mechanism is impaired in aged animals, which may cause the previously described pro-oxidative and proinflammatory state in the nigra of aged brains and the higher vulnerability of dopaminergic neurons with aging. IGF-1 therapy produced some controversial results, possibly because IGF-1 levels adequate to specific situations are critical for health.

## MATERIALS AND METHODS

### Experimental design

In a first series of experiments, we used immunohistochemistry and laser confocal microscopy to detect the presence of IGF-1 and IGF-1R in the different types of cells used in the study (i.e. dopaminergic neurons, astrocytes and microglia, the dopaminergic neuron cell line MES 23.5 and the N9 microglial cell line). We conducted a second series of studies to examine the neuroprotective or neurotoxic effects of AII and IGF-1 on the dopaminergic neuron death induced by the dopaminergic neurotoxin MPP^+^ in primary mesencephalic cultures and the dopaminergic neuron cell line MES 23.5. In a third series of experiments we investigated the effects of increased IGF-1 levels on RAS activity (levels of AT1, AT2 and angiotensinogen) and on major markers of the microglial M1 and M2 phenotypes. We investigated the *in vitro* effects of treatment with IGF-1 on primary mesencephalic neuron-glia cultures, the dopaminergic neuron cell line MES 23.5, the N9 microglial cell line, and astrocyte-enriched primary mesencephalic cultures.

In a fourth series experiments we investigated the effects of increased RAS activity on IGF-1 and IGF-1R levels. We investigated the *in vitro* effects of AII and AT1 and AT2 antagonists and agonists on IGF-1 and IGF-1R levels, and on major markers of the microglial M1 and M2 phenotypes. We used primary mesencephalic cultures, cultures of the dopaminergic neuron cell line MES 23.5, primary cultures lacking microglial cells, and the N9 microglial cell line. We have previously observed that AII exerts pro-oxidative and pro-inflammatory effects via the AII/AT1/NADPH-oxidase pathway thus generating superoxide; for review, see [26, 36]. In the present study, we therefore also investigated the effect of the pro-oxidant compound pyrogallol and the antioxidant compound tempol on levels of IGF-1 in the above-mentioned cultures.

In the *in vivo* experiments young adult rats were treated with intraventricular injection of AII to investigate the effects of increased AII levels on IGF-1 levels in the nigral region. The rats were injected in the third ventricle (stereotaxic coordinates: 0.8 mm posterior to bregma, midline, 6.5 mm ventral to the dura, and tooth bar at 0) with a single injection of AII (5μg in 3 μl of sterile saline, *n* = 8; Sigma). Control rats were injected with vehicle (*n* = 6 per group). The solution was injected, at a rate of 0.5 μl/min, with a 10 μl Hamilton syringe coupled to a motorized injector (Stoelting). Four or sixteen hours after the AII injection the rats were killed and processed for western blotting (WB). The effective doses of AII and survival periods were determined on the basis of our previous findings [44, 53]. As RAS activity in the nigral region is known to be higher in aged rats than in young rats (i.e. increased activity in the AII/AT1/NADPH-oxidase pathway [34], we also investigated the levels of IGF-1 in aged rats (*n* = 11; 20 moths of age) relative to young rats (*n* = 11; 2.5 months of age). The brains were rapidly removed and the mesencephalon was cut coronally (1 mm) with a tissue chopper. To isolate the nigral region the individual 1mm tissue slides were dissected on a pre-cooled glass plate under a stereoscopic microscope. The substantia nigra compacta (SNc) was dissected according to Paxinos [54], frozen on dry ice, and stored at −80ºC until being processed for WB studies (see below). Rats were housed under a 12 h light/dark cycle with *ad libitum* access to food and water. All experiments were carried out in accordance with EU Directive 2010/63 and EEC Directive 86/609 and were approved by the corresponding committee at the University of Santiago de Compostela. All rats were anaesthetized with ketamine/medetomidine prior to undergoing surgery.

### Primary mesencephalic cultures. N9 microglial and MES 23.5 dopaminergic neuron cell line cultures

Ventral mesencephalic tissue was dissected from rat embryos of 14 days gestation (E14). The tissue was processed as described in our previous studies [43, 44]. The primary neuron-glia cultures were maintained in a humidified CO_2_ incubator (5% CO_2_; 37°C) for 7 days *in vitro* (DIV); the medium was totally removed on day 2 and replaced with fresh culture medium. To obtain cultures lacking microglial cells, L-leucine methyl ester (LME; 1.5 mM; Sigma) was added 48 h after seeding the cells and was maintained in the cultures for 72 h to deplete microglia. This method yields primary mesencephalic cultures containing < 0.1% microglia [55]. For primary astrocytic cultures, cells were incubated (37°C, 5% CO_2_) until confluent; the medium was totally removed on day 2 and replaced with fresh culture medium. After 3 days of incubation, culture dishes were agitated at 180 rpm for 14 h at 30°C. The supernatant containing microglia and neurons was discarded leaving only astrocytes adhered to the dish surface. Astrocytes were dislodged by incubation with trypsin and split into 12-well plates and incubated for approximately 12 days before experiments were conducted. This method can enrich astrocytes to > 97% purity.

The murine N9 microglial cell line was provided by Dr Paola Ricciardi-Castagnoli (Singapore Immunology Network, Agency for Science, Technology and Research, Singapore). The N9 microglial cells were cultured in Roswell Park Memorial Institute medium (RPMI 1640; Invitrogen, 21,875-091) supplemented with 10% FBS, 2mM-Glutamine (Sigma, G6392), 100 U/ml penicillin, and 100 mg/ml streptomycin. The cultures were maintained at 37ºC, 95% air, and 5% CO_2_ in a humidified incubator [56]. The cells were then seeded onto 35-mm culture dishes (0.5 × 10^6^ cells/ well) for analysis.

Dopaminergic MES 23.5 cells, a gift from Dr Wei-dong Le (Baylor College of Medicine, TX, USA), were cultured in DMEM/F12 containing Sato's components growth medium supplemented with 2% fetal bovine serum (FBS), 100 units/ml penicillin, and 100 mg/ml streptomycin at 37 ºC in a humidified CO_2_ incubator (5% CO_2_, 95% air) [57]. For experiments, MES 23.5 cells were plated at a density of 0.5 × 10^5^/cm^2^ onto 35-mm plastic dishes, glass coverslips, previously coated with poly-L-ornithine (P-4638, Sigma; 10 mg/ml). Cells were stimulated to enhance differentiation by adding dibutyryl-cAMP (D0627, Sigma; 1 mM) to the supplemented growth medium, and were grown to 80% confluence before treatment.

### Treatment of cultures

Cultures in the second series of experiments were used to study the effects of AII and IGF-1 on cell loss induced by the DA neurotoxin MPP^+^. Cultures were exposed on 4 DIV to MPP^+^ alone (0.25 μM for primary cultures or 10 μM for the MES 23.5 dopaminergic cell line; Sigma) or to MPP^+^ plus AII (100nM) or MPP^+^ plus IGF-1 (100nM) for a further 4 days. The cultures were processed for WB or fixed with 4% paraformaldehyde and processed for immunohistochemistry against tyrosine hydroxylase (TH; see below). Cultures in the third series of experiments were exposed to IGF-1 (50 and 100 nM; Sigma) to investigate the effects on RAS components and major markers of the microglial M1 and M2 phenotypes. Cultures in the fourth series of experiments were treated with AII (100 nM) for 24 h to investigate effects on levels of IGF-1 or IGF-1R, and major markers of the microglial M1 and M2 phenotypes. The most effective dose of AII was determined on the basis of our previous findings [28, 29]. Some cultures were treated with the AT1 receptor antagonist ZD-7155 (1 μM; Tocris) or the AT2 receptor antagonist PD-123319 (1 μM; Sigma) or the NF-κB inhibitor PDTC (50μM; Sigma) for 30 minutes before treatment with AII to confirm the involvement of AT1 or AT2 receptors. Other cultures were treated with the pro-oxidant compound pyrogallol (50 μM; 24h; Sigma) or the antioxidant tempol (1mM; Sigma) to study effects on IGF-1 levels. The cells were then washed and processed for WB or RT-PCR.

### Western blotting of cell cultures and rat brains

Tissue from rat ventral midbrain and cultured cells (primary mesencephalic cultures, MES 23.5 cells, N9 cells) were lysed in RIPA buffer containing protease inhibitor cocktail (Sigma) and PMSF (Sigma). Cell and tissue lysates were centrifuged, and the protein concentrations were determined by the BCA protein assay (Pierce). Equal amounts of protein were separated by 10% Bis-Tris polyacrylamide gel, and transferred to nitrocellulose membranes. The membranes were incubated overnight with the following primary antibodies: goat anti-AT1 (sc-31181), rabbit anti-AT2 (sc-9040), goat anti-Angiotensin precursor (sc-7419), goat anti-IGF-1 (sc-7144), rabbit anti- IGF-1R (sc-713) from Santa Cruz Biotechnology Inc., 1:200; rabbit anti-iNOS (ab3523, 1:1500) and goat anti-arginase-1 (ab60176, 1:1500) from Abcam; and mouse monoclonal anti-tyrosine hydroxylase (T2928; 1:10000) from Sigma. The specificity of the antibodies was established in previous studies: AT1 sc-31181 [58], AT2 sc-9040 [59, 60], angiotensin precursor sc-7419 [61], IGF-1 sc-7144 [62], IGF-1R sc-713 [63, 64], iNOS ab3523 [65], and arginase-1 ab60176 [66]. In addition, the specificity of AT1 and AT2 antibodies was confirmed in our laboratory by preadsorption with the corresponding synthetic peptide antigen [67] and western blot analysis of lysates from HEK293 cells transfected with AT1 or AT2 tagged to fusion tail DDK (TA50011 from Origene; DDK tag: DYKDDDDK). The specify of the antibodies was confirmed by the presence of a predominant immunoreactive band in positively transfected lysates and the absence of this band in negative controls, which consisted of lysates transfected with empty vectors. Then membranes were treated with the corresponding HRP conjugated secondary, and immunoreactivity was detected with an Immun-Star HRP Chemiluminescent Kit (170-5044, Bio-Rad) and visualized with a chemiluminescence detection system (Molecular Imager ChemiDoc XRS System, Bio-Rad). Blots were reprobed for anti-GAPDH (Sigma; 1:50000) or α-tubulin (Sigma; 1:50,000) as a loading control. In each sample, protein expression was measured by densitometry of the corresponding band and was expressed relative to the GAPDH α-tubulin band value. The data were then normalized to the values of the control group of the same batch (i.e., they were expressed relative to the value obtained for the control; 100%) to counteract any inter-batch variability. Finally, the results were expressed as means ± SEM.

### RNA extraction and real-time quantitative polymerase chain reaction

Total RNA from the nigral region was extracted with Trizol (Invitrogen), according to the manufacturer's instructions. Total RNA (2.5 mg) was reverse-transcribed to complementary DNA with nucleoside triphosphate containing deoxyribose, random primers, and Moloney murine leukemia virus reverse transcriptase (200U; Invitrogen). Real-time PCR was used to examine the relative levels of AT1, AT2, angiotensinogen and IGF-1. Experiments were performed with a real-time iCyclerTM PCR platform (BioRad). β-Actin was used as housekeeping gene and was amplified in parallel with the genes of interest. The comparative cycle threshold values (Ct) method was used to examine the relative mRNA expression. Expression of the genes was determined relative to the housekeeping transcripts. The data were then normalized to the values of the control group of the same batch to counteract any variability between batches. Finally, the results were expressed as mean values ± SEM. Primer sequences were as follows: for AT1, forward 5′-GCTAGGCAATAGTCATCAAC-3′, reverse 5′- GAGAGAATCACAGCAGTTTG-3′; for AT2, forward 5′-CTGGCAAGCATCTTATGTAGTTC-3′, reverse 5′ CAAGCATTCACACCTAAGTATTCA-3′; for angiotensinogen, forward 5′-GAGTGAGGCAAGAGGTGTA-3′, reverse 5′-TCCAACGATCCAAGGTAGAA-3′; for IGF-1, forward 5′-TGTGACATTGCTCTAACATCTC-3′, reverse 5′-GTTGGAAGGCTGCTGATT-3′; and for β-actin, forward 5′-TCGTGCGTGACATTAAAGAG-3′, reverse 5′-TGCCACAGGATTCCATACC-3′

### ELISA

Cultured cells (N9 microglial cells) were homogenized in RIPA buffer containing protease inhibitor cocktail (Sigma) and PMSF (Sigma). The homogenates were centrifuged, at 12,000 g for 20 minutes at 4ºC, and the protein concentrations were determined by the BSA protein assay protein assay (Pierce). The levels of TNF-α were quantified with mice-specific enzyme-linked immunosorbent assay (ELISA) kits according to the manufacturers' instructions (murine TNF-α ELISA kit from Diaclone, 860.040.192). The TNF-α levels in culture samples were obtained in pg per milliliter protein.

### Double immunofluorescence and TH-immunohistochemistry of cell cultures

Cultures used for double immunofluorescence analysis were grown on glass coverslips and fixed with 4% paraformaldehyde (PFA) in Dulbecco's phosphate buffered saline (DPBS; pH 7.4) for 20 min. The cultures were subsequently processed for double fluorescent labelling and incubated overnight at 4°C with the corresponding primary antibodies diluted in DPBS-1% bovine serum albumin (BSA) with 2% normal donkey serum (Sigma). The following primary antibodies were used: rabbit mouse monoclonal anti tyrosine hydroxylase (TH; T2928, Sigma, 1:5000), as a marker of dopaminergic neurons; mouse anti CD11b (complement receptor-3, clone MRC OX42, Serotec; 1:50) as a marker of microglial cells; mouse anti glial fibrillary acidic protein (GFAP, Millipore, 1:500), as a marker of astrocytes; goat anti IGF-1 (sc-7144; Santa Cruz Biotechnology Inc.) as marker of IGF-1 and rabbit anti-IGF-1R (sc-713; Santa Cruz) for IGF-1R. The cultures were rinsed with DPBS before being incubated for 2h with the corresponding fluorescent secondary antibodies. Colocalization of markers was confirmed by confocal laser microscopy (A0-SP5XB5; Leica) and use of a sequential scanning method to prevent overlap. The relative intracellular levels of IGF-1 and IGF-1R were estimated by computer-assisted fluorescence intensity measurements as previously described [53]. Briefly, cell cultures were doubly labeled for IGF-1 or IGF-1R and different cellular markers (TH, GFAP or OX-42). Images from at least 30 cells per group were obtained by laser scanning microscopy at 63 × objective using constant microscope parameters and similar laser intensity. The labelling intensity of IGF-1 and IGF-1R immunofluorescence was measured in individual cells by using the LAS AF Lite software (Leica). Only cell profiles including sectioned nucleus were included, and the background intensity from each image was subtracted from the assessed immunofluorescence intensity of each individual cell. The data were normalized to the value a control group (100%) and they were expressed as mean ± SEM.

Effects of IGF-1 and AII on dopaminergic neuron death induced by MPP^+^ (second series of experiments) were determined by TH-immunohistochemistry using mouse anti-TH (T2928, 1:30,000; Sigma) as described in previous studies [28, 38]. Cells were counted in 5 randomly chosen longitudinal and transverse microscopic fields along the diameter of the culture dish away from the curved edge by an operator who was blind to the treatment condition. The microscopic field was defined by a 0.5 × 0.5-cm reticule (1.25 cm^2^). The average number of TH-positive cells in a control culture dish was 4198 ± 395. The final results were obtained from at least 3 separate experiments, with a minimum sample size of 4 wells per group and per run. The results were expressed as percentages of the counts of the control group in the same batch to counteract possible variations among batches.

### Statistical analysis

All data were obtained from at least three independent experiments and were expressed as mean values ± SEM. Two-group comparisons were carried out by Student's t test and multiple comparisons by one-way ANOVA followed by the Holm Sidak test. The normality of populations and homogeneity of variances were tested before each ANOVA. Differences were considered statistically significant at *p* = 0.05. Statistical analyses were carried out with SigmaStat 3.0 (Jandel Scientific, San Rafael, CA, USA).

## Abbreviations

AII: Angiotensin-II
ARG-1: Arginase-1
AT1: AII type 1 receptor
AT2: AII type 2 receptor
DIV: Days in vitro
FBS: Fetal bovine serum
GFAP: Glial fibrillary acidic protein
IGF-1: Insulin-like growth factor 1
IGF-1R: IGF-1 receptors
LME: L-leucine methyl ester
OS: Oxidative stress
PD: Parkinson's disease
PDTC: Ammonium pyrrolidinedithiocarbamate, NF-κB inhibitor
RAS: Renin-angiotensin system
ROS: Reactive oxygen species
RT-PCR: Real-time quantitative reverse transcription polymerase chain reaction
SNc: Substantia nigra compacta
TH: Tyrosine hydroxylase
WB: Western blotting
